# Long-Term Exposure to Silica Dust and Risk of Total and Cause-Specific Mortality in Chinese Workers: A Cohort Study

**DOI:** 10.1371/journal.pmed.1001206

**Published:** 2012-04-17

**Authors:** Weihong Chen, Yuewei Liu, Haijiao Wang, Eva Hnizdo, Yi Sun, Liangping Su, Xiaokang Zhang, Shaofan Weng, Frank Bochmann, Frank J. Hearl, Jingqiong Chen, Tangchun Wu

**Affiliations:** 1 Department of Occupational and Environmental Health, School of Public Health, Tongji Medical College, Huazhong University of Science and Technology, Wuhan, Hubei, China; 2 Ministry of Education Key Lab of Environment and Health, School of Public Health, Tongji Medical College, Huazhong University of Science and Technology, Wuhan, Hubei, China; 3 Division of Respiratory Disease Studies, National Institute for Occupational Safety and Health, Centers for Disease Control and Prevention, Morgantown, West Virginia, United States of America; 4 Department of Applied Epidemiology, Institute for Occupational Safety and Health, German Social Accident Insurance (IFA), Sankt Augustin, Germany; 5 Daye Iron Mine Hospital, Wuhan Iron and Steel Corporation, Huangshi, Hubei, China; 6 Jingdezhen Health Bureau, Jingdezhen, Jiangxi, China; University of Pittsburgh School of Medicine, United States of America

## Abstract

**Background:**

Human exposure to silica dust is very common in both working and living environments. However, the potential long-term health effects have not been well established across different exposure situations.

**Methods and Findings:**

We studied 74,040 workers who worked at 29 metal mines and pottery factories in China for 1 y or more between January 1, 1960, and December 31, 1974, with follow-up until December 31, 2003 (median follow-up of 33 y). We estimated the cumulative silica dust exposure (CDE) for each worker by linking work history to a job–exposure matrix. We calculated standardized mortality ratios for underlying causes of death based on Chinese national mortality rates. Hazard ratios (HRs) for selected causes of death associated with CDE were estimated using the Cox proportional hazards model. The population attributable risks were estimated based on the prevalence of workers with silica dust exposure and HRs. The number of deaths attributable to silica dust exposure among Chinese workers was then calculated using the population attributable risk and the national mortality rate. We observed 19,516 deaths during 2,306,428 person-years of follow-up. Mortality from all causes was higher among workers exposed to silica dust than among non-exposed workers (993 versus 551 per 100,000 person-years). We observed significant positive exposure–response relationships between CDE (measured in milligrams/cubic meter–years, i.e., the sum of silica dust concentrations multiplied by the years of silica exposure) and mortality from all causes (HR 1.026, 95% confidence interval 1.023–1.029), respiratory diseases (1.069, 1.064–1.074), respiratory tuberculosis (1.065, 1.059–1.071), and cardiovascular disease (1.031, 1.025–1.036). Significantly elevated standardized mortality ratios were observed for all causes (1.06, 95% confidence interval 1.01–1.11), ischemic heart disease (1.65, 1.35–1.99), and pneumoconiosis (11.01, 7.67–14.95) among workers exposed to respirable silica concentrations equal to or lower than 0.1 mg/m^3^. After adjustment for potential confounders, including smoking, silica dust exposure accounted for 15.2% of all deaths in this study. We estimated that 4.2% of deaths (231,104 cases) among Chinese workers were attributable to silica dust exposure. The limitations of this study included a lack of data on dietary patterns and leisure time physical activity, possible underestimation of silica dust exposure for individuals who worked at the mines/factories before 1950, and a small number of deaths (4.3%) where the cause of death was based on oral reports from relatives.

**Conclusions:**

Long-term silica dust exposure was associated with substantially increased mortality among Chinese workers. The increased risk was observed not only for deaths due to respiratory diseases and lung cancer, but also for deaths due to cardiovascular disease.

*Please see later in the article for the Editors' Summary*

## Abstract 2

A retro-prospective cohort study by Weihong Chen and colleagues provides new estimates for the risk of total and cause-specific mortality due to long-term silica dust exposure among Chinese workers.

## Editors' Summary

**Background:**

Walk along most sandy beaches and you will be walking on millions of grains of crystalline silica, one of the commonest minerals on earth and a major ingredient in glass and in ceramic glazes. Silica is also used in the manufacture of building materials, in foundry castings, and for sandblasting, and respirable (breathable) crystalline silica particles are produced during quarrying and mining. Unfortunately, silica dust is not innocuous. Several serious diseases are associated with exposure to this dust, including silicosis (a chronic lung disease characterized by scarring and destruction of lung tissue), lung cancer, and pulmonary tuberculosis (a serious lung infection). Moreover, exposure to silica dust increases the risk of death (mortality). Worryingly, recent reports indicate that in the US and Europe, about 1.7 and 3.0 million people, respectively, are occupationally exposed to silica dust, figures that are dwarfed by the more than 23 million workers who are exposed in China. Occupational silica exposure, therefore, represents an important global public health concern.

**Why Was This Study Done?:**

Although the lung-related adverse health effects of exposure to silica dust have been extensively studied, silica-related health effects may not be limited to these diseases. For example, could silica dust particles increase the risk of cardiovascular disease (diseases that affect the heart and circulation)? Other environmental particulates, such as the products of internal combustion engines, are associated with an increased risk of cardiovascular disease, but no one knows if the same is true for silica dust particles. Moreover, although it is clear that high levels of exposure to silica dust are dangerous, little is known about the adverse health effects of lower exposure levels. In this cohort study, the researchers examined the effect of long-term exposure to silica dust on the risk of all cause and cause-specific mortality in a large group (cohort) of Chinese workers.

**What Did the Researchers Do and Find?:**

The researchers estimated the cumulative silica dust exposure for 74,040 workers at 29 metal mines and pottery factories from 1960 to 2003 from individual work histories and more than four million measurements of workplace dust concentrations, and collected health and mortality data for all the workers. Death from all causes was higher among workers exposed to silica dust than among non-exposed workers (993 versus 551 deaths per 100,000 person-years), and there was a positive exposure–response relationship between silica dust exposure and death from all causes, respiratory diseases, respiratory tuberculosis, and cardiovascular disease. For example, the hazard ratio for all cause death was 1.026 for every increase in cumulative silica dust exposure of 1 mg/m^3^-year; a hazard ratio is the incidence of an event in an exposed group divided by its incidence in an unexposed group. Notably, there was significantly increased mortality from all causes, ischemic heart disease, and silicosis among workers exposed to respirable silica concentrations at or below 0.1 mg/m^3^, the workplace exposure limit for silica dust set by the US Occupational Safety and Health Administration. For example, the standardized mortality ratio (SMR) for silicosis among people exposed to low levels of silica dust was 11.01; an SMR is the ratio of observed deaths in a cohort to expected deaths calculated from recorded deaths in the general population. Finally, the researchers used their data to estimate that, in 2008, 4.2% of deaths among industrial workers in China (231,104 deaths) were attributable to silica dust exposure.

**What Do These Findings Mean?:**

These findings indicate that long-term silica dust exposure is associated with substantially increased mortality among Chinese workers. They confirm that there is an exposure–response relationship between silica dust exposure and a heightened risk of death from respiratory diseases and lung cancer. That is, the risk of death from these diseases increases as exposure to silica dust increases. In addition, they show a significant relationship between silica dust exposure and death from cardiovascular diseases. Importantly, these findings suggest that even levels of silica dust that are considered safe increase the risk of death. The accuracy of these findings may be affected by the accuracy of the silica dust exposure estimates and/or by confounding (other factors shared by the people exposed to silica such as diet may have affected their risk of death). Nevertheless, these findings highlight the need to tighten regulations on workplace dust control in China and elsewhere.

**Additional Information:**

Please access these websites via the online version of this summary at http://dx.doi.org/10.1371/journal.pmed.1001206.

- The American Lung Association provides information on silicosis
- The US Centers for Disease Control and Prevention provides information on silica in the workplace, including links to relevant US National Institute for Occupational Health and Safety publications, and information on silicosis and other pneumoconioses
- The US Occupational Safety and Health Administration also has detailed information on occupational exposure to crystalline silica
- What does silicosis mean to you is a video provided by the US Mine Safety and Health Administration that includes personal experiences of silicosis; Dont let silica dust you is a video produced by the Association of Occupational and Environmental Clinics that identifies ways to reduce silica dust exposure in the workplace
- The MedlinePlus encyclopedia has a page on silicosis (in English and Spanish)
- The International Labour Organization provides information on health surveillance for those exposed to respirable crystalline silica
- The World Health Organization has published a report about the health effects of crystalline silica and quartz

## Introduction

Crystalline silica is one of the most ubiquitous minerals on earth, with widespread exposure in working and living environments. Multiple serious diseases and increased mortality have been associated with exposure to crystalline silica, making it a high-priority public health concern. Occupational silica exposure and its related health effects rank among the most important public health concerns in developing and developed nations. Recent reports indicate that more than 23 million workers are exposed to crystalline silica in China [1] and over 10 million in India alone [2]. In the United States and Europe, the respective figures are 1.7 million [3] and over 3 million [4]. Silica dust is generated at industrial sources and transported to environments, and it is also generated by such natural phenomena as volcanic explosions and sandstorms.

Adverse health effects from exposure to silica dust are of increasing public health concern worldwide, and have been studied for many years [5]. Silicosis is a well known consequence of silica dust exposure, and exposure has also been associated with the risk of lung cancer, pulmonary tuberculosis, and other airway diseases [6]–[9]. However, silica-related health effects are not limited to those diseases [10]. The potential health effects of particulate exposure on cardiovascular diseases (CVDs) have drawn recent attention, but have yet to be well studied in workers exposed to silica dust. Several studies suggest that ambient particulates (mainly combustion-sourced) are associated with an elevated risk of death [11]–[13] and CVD [14],[15]. Silica is a non-combustion-sourced particle, but its role in the pathogenesis of CVD also needs to be addressed. In addition, adverse health effects from low levels of silica exposure (below legally set exposure limits) need further evaluation.

Therefore, we present results from a retro-prospective cohort study of 74,040 Chinese workers followed from January 1, 1960, to December 31, 2003. Cumulative silica dust exposure (CDE) was calculated for each worker using a job–exposure matrix (JEM) based on a large number of measurements broken down by job title and collected since 1950. Our objectives were to quantify the health effects of silica exposure on cause-specific mortality and to determine population attributable risks (PARs) of mortality associated with the exposure in Chinese workers.

## Methods

### Study Population and Health Data

We identified 74,040 workers from 20 metal mines and nine pottery factories in central and southern China. All individuals were unrelated ethnic Han Chinese. We selected workplaces with systematically collected data on silica dust exposure and workers' health condition. The study included ten tungsten mines in Jiangxi and Hunan provinces, six iron and copper mines in Hubei province, four tin mines in Guangxi province, and nine pottery factories in Jiangxi, Hunan, and Henan provinces (Figure S1). The cohort included all 74,040 workers who were registered in company employment records—which included personnel files, individual medical records, occupational records, and wage rosters—for at least 1 y between January 1, 1960, and December 31, 1974. We collected retrospective data on vital status, work history, and newly diagnosed pneumoconiosis (silicosis) until 1986, with mortality follow-up until the end of 2003.

Trained investigators used a questionnaire to collect data on demographic information, cigarette use, and drinking habits since 1986. In 2004, occupational history and other updates were collected from survivors or those still employed. We defined positive silica dust exposure status as employment in a silica-dust-exposed job for 6 mo or more. Work histories for silica-dust-exposed workers were taken from company occupational records. Data included job titles, work start and end dates, and reasons for leaving (e.g., retirement or workplace change).

All individuals were tracked for their vital status by local hygienists (or occupational health doctors) from January 1, 1960, through December 31, 2003. We classified cause of death evidence by levels of confidence in the data: Level 1—medical record from a hospital or a personal doctor at a local hospital (60.5%); Level 2—cause of death recorded in an employment register, accident record, or death certificate (35.2%); and Level 3—oral reports from relatives (4.3%). Results did not change materially after excluding Level 3 deaths. We used the 10th International Classification of Diseases (ICD-10) to code causes of death.

All workers exposed to silica dust received chest radiographs every 2 to 4 y, even after cessation of dust exposure. National diagnostic criteria for pneumoconiosis were standardized as stage I, II, or III. These categories have been previously described [16]. The study was approved by the Tongji Medical College Institutional Review Board and the US National Institute for Occupational Safety and Health Institutional Review Board.

### Occupational Exposure Data

We conducted a detailed quantitative occupational exposure evaluation using data from historical industrial health records. Industrial health record-keeping for occupational hazards in each mine or factory started in the early 1950s, when the Chinese government enforced systematic dust sampling regulations that required monthly measurement of dust concentrations in workplaces. The dust monitoring scheme involved measuring total airborne dust concentration by a gravimetric method for each dust-exposed job title, and using a microscopic sizing method to determine particle size distribution and crystalline silica content (quartz by X-ray diffraction method) in bulk samples of settled dust [17].

For the purpose of this study, more than 4,200,000 environmental measurements of total dust concentrations from 29 mines and factories from 1950 to 2003 were used to create a JEM. In this matrix, the total dust concentrations associated with each job title were averaged by year, then listed, along with specific facility and job titles, for each calendar year [18]. For missing data for years or jobs (less than 20%), total dust concentrations were estimated using monitoring data for similar jobs or for the same job at different times. In the matrix, there were 1,090 facility–job title combinations for 54 calendar-year periods from 1950 to 2003.

We used the JEM of total dust concentrations to estimate silica dust exposure for each worker. In this matrix, total silica dust concentrations were listed along with specific facility and job titles for each calendar year. We used all available total dust concentrations for each job to create this JEM. The results indicated good agreement for measured total dust concentrations (*r* ^2^ = 0.84) between Chinese and US methods [19]. To convert the total dust JEM into a respirable silica JEM, each respirable silica concentration was estimated by total dust concentration multiplied by a conversion factor. The conversion factors from Chinese total dust to US respirable silica concentrations (quartz by X-ray diffraction method) were developed based on paired side-by-side dust measurements. The conversion factors of respirable silica concentration to total dust concentration were estimated to be 0.0143 for iron/copper mines, 0.0355 for pottery factories, 0.0429 for tin mines, and 0.0861 for tungsten mines [17]. These conversion factors were updated in a recent analysis with additional data from the same side-by-side measurements conducted from September 1, 2003, to June 30, 2009. The new analysis confirmed that the mean crystalline silica percentage of total dust measurements did not change substantially over time.

Complete work histories for each study individual were taken from personal employment records in mine/factory files. Work histories include job titles and calendar years for each worker's full duration of employment. They were used with the JEM to estimate CDE for individual workers as follows:
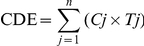
where CDE is cumulative respirable silica dust exposure in milligrams/cubic millimeter–years; *n* is the total number of job titles held by the individual during his or her work history; *C_j_* is 8-h time-weighted mean concentrations of dust in milligrams/cubic millimeter for the *j*th job title within a facility and employment period; and *T_j_* is duration of employment in years in the *j*th job. We calculated CDE from the starting date of dust-exposed work until employees were either lost to follow-up, ended employment, or died.

We used data from a standardized monitoring program in all industrial facilities to track potential environmental hazards, including radon, polycyclic aromatic hydrocarbons, asbestos, talc, and metal elements. Findings indicated very low exposure to asbestos, nickel, talc, and cadmium in the studied workplaces.

### Statistical Analysis

We used Cox proportional hazards regressions to estimate the hazard ratios (HRs) and 95% confidence intervals (CIs) for selected causes of death by different levels of CDE compared with no exposure. CDE was categorized into low, medium, and high levels based on equally spaced percentiles from the exposure distribution in the entire cohort. Further, tests of linear trend were conducted by including the median value for each level of CDE as a continuous variable in the models. We also estimated the HRs associated with a 1 mg/m^3^-y increase in CDE by entering CDE into the models as a continuous variable. In addition, nonlinear association was assessed by adding a quadratic term (CDE and square of CDE, continuous) to the model. Other covariates included in the model were gender, year of hire (five categories, 1955 or earlier, 1956–1960, 1961–1965, 1966–1970, and 1970 or later), age at hire (continuous), and type of mine/factory (four categories, tungsten, iron/copper, tin, and pottery) as potential confounders. For mortality with possible nonlinear associations, we further examined the detailed exposure–response relationship of mortality risk across the range of CDEs using a penalized spline regression model [20]. The sample size (2.3 million person-years) was too large for fitting a Cox proportional hazards model with penalized splines; therefore, we created a nested case–control sample for each specific cause of death by randomly selecting 20 controls (matched for type of mine/factory and gender) for each case who were alive at the time of the case's death. The penalized spline regression model was fitted with and without adjustment for smoking (never smoked/ever smoked) to detect the potential confounding effect of smoking. The sensitivity of the model was tested by selecting different degrees of freedom and excluding influential outliers.

The PAR was calculated with the following equation:
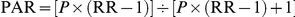
where *P* is the prevalence of silica-exposed workers among all industrial workers (16.3%) [1], and RR is the multivariate-adjusted relative risk. The HRs from Cox proportional hazards models of this cohort were used as estimates of RR.

Standardized mortality ratios (SMRs) were defined as the ratio of observed to expected deaths [21]. National death rate data were not available before 1970; therefore, individuals who died before that time were not included in the SMR analysis. In total, SMRs were calculated using 17,783 deaths. We calculated the expected number of cause-specific deaths by multiplying the gender-, age-, period-, and cause-specific person-years at risk (5-y intervals for age and period) by the corresponding mortality rates in the Chinese national population [22]. We obtained the 95% CIs for SMRs by setting limits for the numerator and the observed number of cases, and by assuming the denominator to be a constant [21]. A *p*-value≤0.05 was considered statistically significant. The penalized spline regression analyses were conducted using S-Plus version 8.0 (Insightful Corporation); all other analyses were performed using SAS version 9.1 (SAS Institute).

## Results

The cohort included 74,040 individuals (63,529 males, 85.8%). The average age was 27.2 y for individuals entering into the cohort. And 16.2% were still working at the end of follow-up (Table S1). The baseline characteristics of the cohort and follow-up information are summarized in Table 1. A total of 49,309 (66.6%) of workers were exposed to silica dust. The largest number of exposed workers (78.9%) worked in tungsten mines; the lowest (48.5%), in iron and copper mines. During a median follow-up period of 33.1 y (2,306,428 person-years), 19,516 deaths were reported. Mortality was 846.2 per 100,000 person-years, with 992.6 per 100,000 person-years among dust-exposed workers and 550.7 per 100,000 person-years among non-dust-exposed workers.

**Table 1:** Description of the cohort (*n* = 74,040) based on different types of mine/factory, 1960–2003.

Type of Mine/Factory	End of Follow-Up	Number of Mines/Factories	Median Period of Follow-Up (Years)	Number of Workers	Number of Workers Exposed to Silica Dust	Number of Pneumoconiosis Cases	Number of Deaths
Tungsten mines	December 31, 1994	4	29.9	13,857	10,787	3,650	3,678
Tungsten mines	December 31, 2003	6	34.7	19,061	15,170	4,238	7,138
Iron and copper mines	December 31, 1989	4	20.2	7,368	4,355	356	438
Iron and copper mines	December 31, 2003	2	35.5	11,214	4,666	406	2,293
Tin mines	December 31, 1994	1	28.9	2,717	1,838	621	548
Tin mines	December 31, 2003	3	35.5	5,526	3,109	466	1,391
Pottery factories	December 31, 1989	1	30.0	826	496	39	178
Pottery factories	December 31, 1994	4	31.7	6,098	4,050	477	1,404
Pottery factories	December 31, 2003	4	38.0	7,373	4,838	742	2,448
Entire cohort		29	33.1	74,040	49,309	10,995	19,516

**Figure 1:**
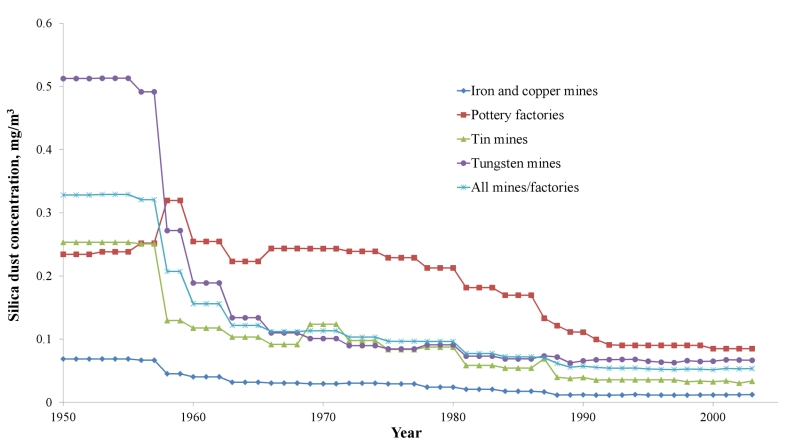
Annual silica dust concentrations, average of all job titles in different mines/factories in China, 1950–2003.

Respirable silica dust levels in the four types of mines/factories from 1960 to the end of 2003 are shown in Figure 1. The mean respirable silica dust concentrations ranged from approximately 0.08 mg/m^3^ in iron mines to 0.52 mg/m^3^ in tungsten mines. Starting in 1960, safer working practices and increased protection measures led to decreased dust concentration and exposure. Mean dust concentration in mines fell to less than 0.1 mg/m^3^ after 1970. Mean dust concentrations in pottery factories were approximately 0.15 to 0.30 mg/m^3^ from the 1960s to the 1980s, and 0.12 to 0.15 mg/m^3^ after 1990.

Table 2 shows the distribution of individuals according to different silica dust exposure levels, the year of birth, age at first hire, CDE, and prevalence of pneumoconiosis. Males accounted for 92.6% of those exposed to silica dust. The prevalence of smokers (current and former) among the entire cohort and the dust-exposed workers was 61.7% (98.7% for males) and 69.4% (99.1% for males), respectively.

**Table 2:** Characteristics of the cohort (*n* = 74,040) based on CDE, 1960–2003.

Characteristic	Entire Cohort (*n* = 74,040)	Levels of CDE^a^			
		Unexposed (*n* = 24,731)	Low (*n* = 15,438)	Medium (*n* = 16,878)	High (*n* = 16,993)
**Number of workers in mines/factories (percent)**					
Tungsten mines	32,918 (44.5)	6,961 (28.1)	7,255 (47.0)	9,626 (57.0)	9,076 (53.4)
Iron and copper mines	18,582 (25.1)	9,561 (38.7)	5,272 (34.1)	2,041 (12.1)	1,708 (10.1)
Tin mines	8,243 (11.1)	3,296 (13.3)	2,082 (13.5)	2,478 (14.7)	387 (2.3)
Pottery factories	14,297 (19.3)	4,913 (19.9)	829 (5.4)	2,733 (16.2)	5,822 (34.3)
**Number of male sex (percent)**	63,529 (85.8)	17,879 (72.3)	14,432 (93.5)	15,659 (92.8)	15,559 (91.6)
**Year of birth**	1937.1±11.1	1939.4±10.9	1941.6±9.6	1935.7±10.2	1930.9±10.5
**Year of birth—number (percent)**					
1900–1919	5,719 (7.7)	1,470 (5.9)	453 (2.9)	1,175 (7.0)	2,621 (15.4)
1920–1929	12,389 (16.7)	2,849 (11.5)	1,459 (9.5)	3,216 (19.1)	4,865 (28.6)
1930–1939	25,441 (34.4)	7,758 (31.4)	4,019 (26.0)	7,108 (42.1)	6,556 (38.6)
1940–1949	20,627 (27.9)	8,064 (32.6)	6,413 (41.5)	3,832 (22.7)	2,318 (13.6)
1950–1963	9,864 (13.3)	4,590 (18.6)	3,094 (20.0)	1,547 (9.2)	633 (3.7)
**Year of hire**	1961.8±7.4	1963.1±7.2	1965.3±6.6	1960.5±6.9	1958.0±6.9
**Year of hire—number (percent)**					
1915–1954	16,181 (21.9)	3,884 (15.7)	969 (6.3)	4,406 (26.1)	6,922 (40.7)
1955–1959	21,383 (28.9)	6,702 (27.1)	3,852 (25.0)	5,683 (33.7)	5,146 (30.3)
1960–1964	8,611 (11.6)	2,933 (11.9)	1,917 (12.4)	2,010 (11.9)	1,751 (10.3)
1965–1969	11,219 (15.2)	4,228 (17.1)	3,197 (20.7)	1,975 (11.7)	1,819 (10.7)
1970–1974	16,646 (22.5)	6,984 (28.2)	5,503 (35.6)	2,804 (16.6)	1,355 (8.0)
**Age at hire (years)**	24.8±7.6	23.9±7.4	23.8±6.2	24.9±7.3	27.2±8.7
**Smoking status** b					
Current smokers—number (percent)	21,438 (48.0)	5,878 (37.9)	5,998 (54.7)	5,397 (57.2)	4,165 (47.5)
Current smokers—pack-years	33.9±17.1	33.1±17.6	33.3±16.7	35.9±17.5	33.2±16.0
Former smokers—number (percent)	6,141 (13.7)	1,458 (9.4)	1,387 (12.6)	1,278 (13.5)	2,018 (23.0)
Former smokers—pack-years	28.5±14.0	30.9±15.6	26.8±13.7	28.5±13.2	27.9±13.3
Never smokers—number (percent)	17,130 (38.3)	8,188 (52.7)	3,587 (32.7)	2,764 (29.3)	2,591 (29.5)
**Duration of silica dust exposure (years)** c	18.7±10.4	0.0±0.0	14.6±9.6	18.1±9.4	23.4±10.3
**CDE (mg/m^3^-y)** c	3.9±4.2	0.0±0.0	0.6±0.3	2.5±0.9	8.5±4.1
**Mean silica dust concentration (mg/m^3^)** c	0.2±0.2	0.0±0.0	0.1±0.1	0.2±0.2	0.4±0.2
**Number of pneumoconiosis cases (percent)** c	10,995 (22.3)	0 (0.0)	678 (4.4)	3,550 (21.0)	6,767 (39.8)
**Year of diagnosis of pneumoconiosis—number (percent)** d					
1955–1959	1,210 (11.0)	NA	19 (2.8)	331 (9.3)	860 (12.7)
1960–1969	4,344 (39.5)	NA	90 (13.3)	1,317 (37.1)	2,937 (43.4)
1970–1979	2,663 (24.2)	NA	198 (29.2)	873 (24.6)	1,592 (23.5)
1980–2003	2,778 (25.3)	NA	371 (54.7)	1,029 (29.0)	1,378 (20.4)
**Age at first diagnosis of pneumoconiosis (years)** d	44.2±10.4	NA	47.8±9.8	43.6±10.9	44.3±10.1
**Latency of pneumoconiosis (years)** d	21.3±10.2	NA	21.1±8.7	18.6±9.7	22.7±10.3

Values expressed as mean ± standard deviation, unless otherwise indicated. Percentages may not total 100 due to rounding.

a Levels are tertiles of CDE of all the workers with exposure to silica dust: low, 0.01–1.23 mg/m^3^-y; medium, 1.24–4.46 mg/m^3^-y; and high, >4.46 mg/m^3^-y.

b Data were available for the sub-cohorts that had been followed through the end of 2003. Smokers were defined as those who had smoked regularly for over 1 y. Smokers who stopped smoking within 1 y before the end of follow-up were defined as current smokers.

c These characteristics were calculated among workers exposed to silica dust. Mean silica dust concentration was calculated as CDE divided by duration of silica dust exposure.

d These characteristics were calculated among workers diagnosed with pneumoconiosis. Latency of pneumoconiosis was defined as the period between the year of first exposure to dust and the year of first diagnosis of pneumoconiosis.

NA, not applicable.

**Figure 2:**
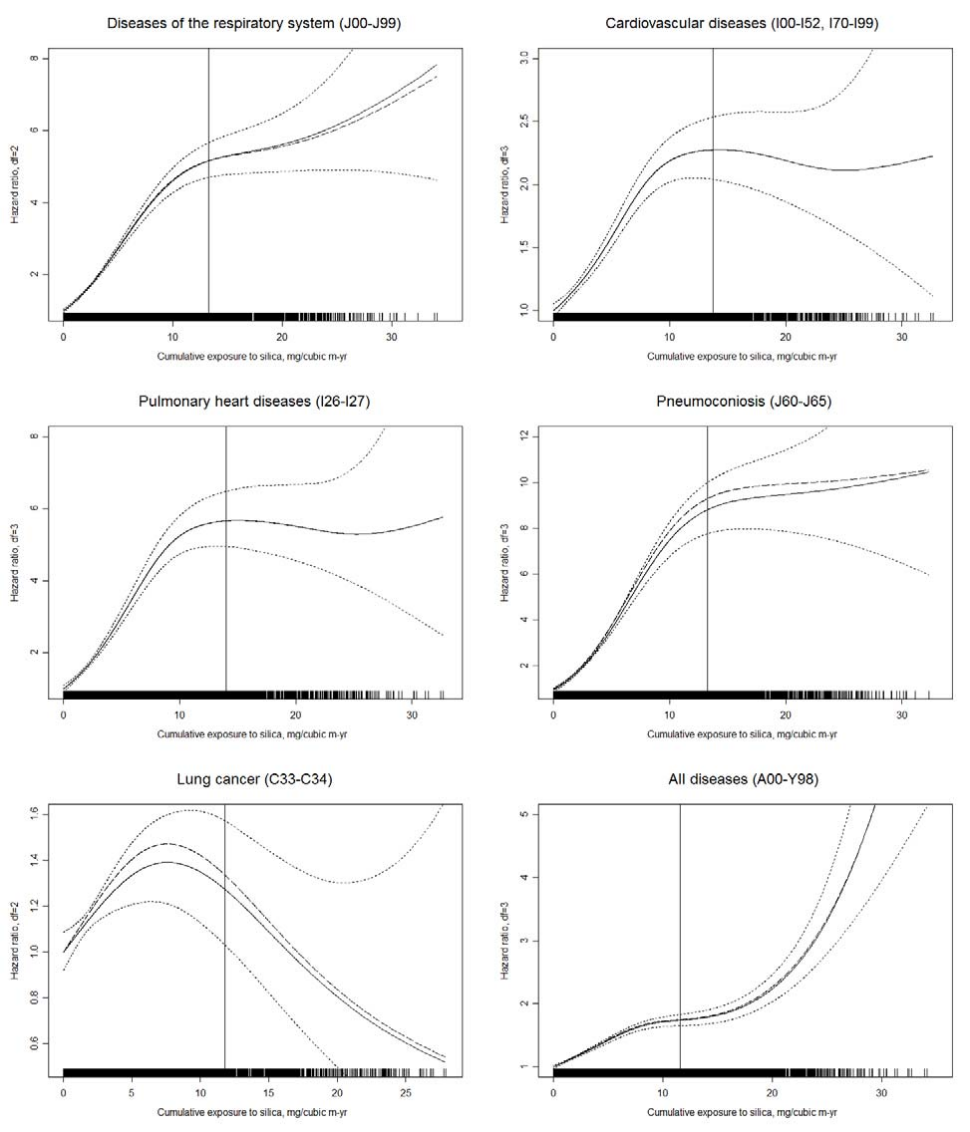
Estimated HRs for total and cause-specific mortality associated with a continuous CDE variable in nested case–control samples from workers with detailed data on historical silica exposure and smoking, 1960–2003. HRs and 95% CIs were derived from penalized spline regression models to examine the nonlinear relation of CDE to mortality. The vertical solid line in each panel represents the 95th percentile of CDE. Dashed lines represent the point estimate of the HR adjusted for duration of follow-up (time-dependent, continuous) and calendar time (time-dependent, continuous); solid lines represent HR further adjusted for smoking (never smoked/ever smoked), with dotted lines indicating the 95% CI; the rug plots along the horizontal axes give the distribution of CDE values. For simplicity of presentation, the reference value of CDE was set to 0 mg/m^3^-y (0.01 mg/m^3^-y for pneumoconiosis).

The numbers of deaths and the HRs for the main mortality causes are shown in Table 3. CVD was the leading cause of death in this cohort. Non-malignant respiratory diseases, malignant neoplasms, infectious diseases, and cerebrovascular disease were the second to fifth causes of death for all cohort members. Mortality from all causes was significantly higher in the group exposed to silica dust compared with the non-exposed group (HR 1.38, 95% CI 1.33–1.43). For both categorical and continuous CDE variables, a positive exposure–response relationship was observed for mortality from all causes, from CVDs (including pulmonary heart disease), from respiratory diseases (including pneumoconiosis), and from infectious diseases (including respiratory tuberculosis). Each 1 mg/m^3^-y increase in CDE was associated with a 2.6%, 6.9%, and 3.1% increase in the mortality risk for all causes, respiratory diseases, and CVDs, respectively. For lung cancer, a positive exposure–response association was observed for categorical CDE (HRs 1.45, 1.53, and 1.46 for low, medium, and high levels of CDE, respectively; *p*-value for linear trend = 0.01); however, the HR for each 1 mg/m^3^-y increase in CDE did not reach statistical significance (HR 1.005, 95% CI 0.987–1.023). The HRs for the association between total and cause-specific mortality and the continuous CDE variable, estimated using penalized spline regressions, are shown in Figure 2. The risk of mortality from all causes increased monotonically with increased CDE; risk of mortality from lung cancer, CVDs (including pulmonary heart disease), and diseases of the respiratory system (including pneumoconiosis) increased monotonically when CDE was lower than about 10 mg/m^3^-y, but became attenuated or even decreased (lung cancer) with higher CDE. Adjustment for smoking slightly attenuated the estimates for lung cancer and pneumoconiosis, but did not change the results for other causes of death (Figure 2).

**Table 3:** Estimated HRs for total and cause-specific mortality associated with CDE in the cohort (*n* = 74,040), 1960–2003.

Cause of Death (ICD-10 Codes)	Number of Events	HR Increase per 1 mg/m^3^-y Increase in CDE	HRs for Levels of CDE versus Unexposed^a^			
			Low	Medium	High	*p*-Value for Trend^b^
**Malignant neoplasms (C00–C97)**	3,621	0.982 (0.972–0.991)	1.20 (1.09–1.32)	1.13 (1.03–1.24)	0.97 (0.88–1.07)	0.06
Malignant neoplasm of nasopharynx (C11)	176	0.942 (0.895–0.991)	0.96 (0.63–1.48)	0.83 (0.55–1.24)	0.58 (0.36–0.93)	0.02
Malignant neoplasm of liver and intrahepatic bile ducts (C22)	1,001	0.972 (0.953–0.991)	1.15 (0.96–1.37)	1.02 (0.86–1.22)	0.87 (0.72–1.06)	0.05
Lung cancer (C33–C34)	949	1.005 (0.987–1.023)	1.45 (1.19–1.75)	1.53 (1.27–1.84)	1.46 (1.19–1.78)	0.01
**Certain infectious and parasitic diseases (A00–B99, J65)**	3,401	1.062 (1.055–1.068)	1.31 (1.09–1.56)	2.70 (2.36–3.08)	3.83 (3.38–4.35)	<0.001
**Respiratory tuberculosis (A15–A16, J65)**	3,100	1.065 (1.059–1.071)	1.30 (1.06–1.60)	3.14 (2.71–3.64)	4.53 (3.94–5.20)	<0.001
**CVDs (I00–I52, I70–I99)**	4,425	1.031 (1.025–1.036)	1.08 (0.96–1.21)	1.42 (1.29–1.57)	1.86 (1.71–2.03)	<0.001
Pulmonary heart diseases (I26–I27)	2,729	1.050 (1.044–1.056)	1.08 (0.88–1.33)	2.32 (2.01–2.67)	3.44 (3.01–3.92)	<0.001
Hypertensive heart disease (I11)	391	0.977 (0.955–0.999)	0.87 (0.62–1.24)	0.83 (0.63–1.11)	0.86 (0.66–1.12)	0.44
Ischemic heart disease (I20–I25)	624	0.971 (0.950–0.994)	1.25 (0.99–1.56)	1.03 (0.82–1.29)	0.80 (0.63–1.02)	0.01
Chronic rheumatic heart disease (I05–I09)	123	0.979 (0.934–1.026)	1.29 (0.74–2.25)	1.16 (0.70–1.92)	0.94 (0.56–1.58)	0.54
**Cerebrovascular diseases (I60–I69)**	2,662	0.997 (0.988–1.006)	1.01 (0.89–1.13)	0.89 (0.79–0.99)	0.90 (0.81–1.00)	0.05
**Diseases of the respiratory system (J00–J99)**	4,309	1.069 (1.064–1.074)	1.89 (1.60–2.24)	4.28 (3.74–4.91)	6.68 (5.85–7.61)	<0.001
Pneumoconiosis (J60–J65)^d^	2,857	1.060 (1.053–1.067)	1.0 (referent)	4.36 (3.49–5.44)	7.75 (6.21–9.67)	<0.001
**Diseases of the digestive system (K00–K93)**	879	0.991 (0.973–1.008)	1.15 (0.94–1.41)	0.88 (0.73–1.08)	0.94 (0.78–1.15)	0.36
**External causes of morbidity and mortality (V01–Y98)**	1,180	0.983 (0.964–1.002)	1.47 (1.25–1.72)	1.17 (0.98–1.39)	1.06 (0.88–1.27)	0.34
**All diseases (A00–Y98)**	19,516	1.026 (1.023–1.029)	1.17 (1.12–1.23)	1.30 (1.25–1.36)	1.58 (1.51–1.64)	<0.001

All Cox proportional hazards models included age as the time variable. Categorical analyses were based on levels of CDE, including unexposed, low, medium, and high; the unexposed level was used as the reference category (low level for pneumoconiosis). In all models, the HRs associated with CDE were adjusted for gender, year of hire (five categories: 1955 or earlier, 1956–1960, 1961–1965, 1966–1970, and 1970 or later), age at hire (continuous), and type of mine/factory (four categories: tungsten, iron/copper, tin, and pottery).

a Levels were tertiles of CDE of all the workers with exposure to silica dust: low, 0.01–1.23 mg/m^3^-y; medium, 1.24–4.46 mg/m^3^-y; and high, >4.46 mg/m^3^-y.

b Assessed by including the median values of exposure within each category as a continuous variable in the model, including the reference category.

For a subset of workers with exposures under the respirable silica concentration limit of 0.1 mg/m^3^ during their lifetime work histories (mean and median CDE were 0.64 and 0.56 mg/m^3^-y, respectively), each 0.1 mg/m^3^-y increase in CDE was associated with a 2.1% (95% CI 1.4%–2.7%), 7.2% (5.2%–9.4%), and 2.4% (0.7%–4.1%) increase in the mortality risk for all diseases, respiratory diseases, and CVDs, respectively. After adjusting for gender, year of hire, age at hire, type of mine/factory, and smoking, the respective mortality risk were 0.8% (0.1%–1.5%), 6.3% (4.1%–8.6%), and 2.2% (0.4%–4.1%) for all diseases, respiratory diseases, and CVDs, respectively; for CVDs, the mortality risk of pulmonary heart disease and ischemic heart disease increased by 6.0% (2.8%-9.3%) and 4.2% (1.4%-7.2%), respectively.

After adjustment for potential confounders including smoking, we estimated the PAR for silica dust exposure. Silica exposure accounted for 15.2% of mortality from all deaths, 63.9% of mortality from respiratory diseases, and 21.0% of mortality from CVDs among the silica-exposed workers. According to an annual health statistical report in China, the prevalence of silica-dust-exposed workers was 16.3% among Chinese industrial workers in 2008 [1]. We estimate that 4.2% of the deaths (231,104) among industrial workers in 2008 were attributable to silica dust exposure based on the relative risks derived from this study.

Table 4 shows the SMRs for deaths from all causes and from the main exposure-related diseases among dust-exposed workers from January 1, 1970, to December 31, 2003. Compared with national mortality in China, workers exposed to silica had significantly elevated mortality from all causes of death (SMR 1.21), and elevated mortality for nasopharynx cancer (1.76), liver cancer (1.16), infectious diseases (6.83), respiratory tuberculosis (4.88), CVDs (1.91), and respiratory diseases (2.32). Among CVDs, mortality for pulmonary heart diseases (4.03) and hypertensive heart disease (2.45) were significantly elevated. For those who worked at annual respirable silica dust concentrations at or below 0.1 mg/m^3^, mortality was significantly elevated for all causes (SMR 1.06, 95% CI 1.01–1.11), pneumoconiosis (11.01, 7.67–14.95), infectious diseases (1.88, 1.55–2.25), and malignant neoplasms (1.10, 1.01–1.19), including nasopharynx cancer (1.63, 1.01–2.40) and liver cancer (1.55, 1.33–1.78). Elevated mortality from CVDs (1.09, 0.97–1.23) included ischemic heart disease (1.65, 1.35–1.99) and hypertensive heart disease (2.53, 1.76–3.44).

**Table 4:** Estimated SMRs for underlying cause of death of silica-dust-exposed workers in the cohort (*n* = 72,248), 1970–2003.

Cause of Death (ICD-10 Codes)	SMR (95% CI)			
	1970 to 1974	1970 to 1984	1970 to 1994	1970 to 2003
**Malignant neoplasms (C00–C97)**	0.69 (0.58–0.82)	0.79 (0.73–0.85)	0.85 (0.81–0.89)	0.82 (0.79–0.85)
Malignant neoplasm of nasopharynx (C11)	2.57 (1.47–3.98)	2.10 (1.57–2.71)	1.80 (1.45–2.19)	1.76 (1.45–2.10)
Malignant neoplasm of liver and intrahepatic bile ducts (C22)	1.06 (0.77–1.38)	1.12 (0.98–1.27)	1.21 (1.11–1.32)	1.16 (1.08–1.25)
Lung cancer (C33–C34)	1.22 (0.74–1.81)	1.00 (0.83–1.17)	0.96 (0.87–1.05)	0.90 (0.84–0.97)
**Certain infectious and parasitic diseases (A00–B99, J65)**	10.40 (9.46–11.38)	7.87 (7.44–8.31)	6.96 (6.66–7.27)	6.83 (6.55–7.11)
**Respiratory tuberculosis (A15–A16, J65)**	4.06 (3.69–4.46)	3.53 (3.33–3.74)	4.47 (4.27–4.68)	4.88 (4.67–5.09)
**CVDs (I00–I52, I70–I99)**	2.25 (2.02–2.49)	1.87 (1.76–1.97)	1.95 (1.87–2.03)	1.91 (1.85–1.98)
Pulmonary heart diseases (I26–I27)	3.49 (3.12–3.88)	2.79 (2.62–2.97)	3.77 (3.60–3.95)	4.03 (3.87–4.20)
Hypertensive heart disease (I11)	0.34 (0.11–0.70)	0.86 (0.63–1.12)	1.70 (1.44–1.98)	2.45 (2.17–2.75)
Ischemic heart diseases (I20–I25)	0.52 (0.19–1.01)	0.83 (0.64–1.04)	0.83 (0.72–0.95)	1.04 (0.94–1.14)
Chronic rheumatic heart diseases (I05–I09)	0.29 (0.13–0.52)	0.32 (0.22–0.46)	0.53 (0.41–0.66)	0.56 (0.45–0.69)
**Diseases of the respiratory system (J00–J99)**	6.52 (5.94–7.12)	3.71 (3.52–3.92)	2.61 (2.51–2.72)	2.32 (2.24–2.40)
Pneumoconiosis (J60–J65)	180.20 (163.32–197.90)	117.11 (110.12–124.31)	97.44 (92.89–102.11)	88.13 (84.38–91.97)
**Diseases of the digestive system (K00–K93)**	0.33 (0.23–0.44)	0.55 (0.48–0.63)	0.75 (0.68–0.82)	0.86 (0.80–0.93)
**External causes of morbidity and mortality (V01–Y98)**	0.95 (0.79–1.14)	1.11 (1.00–1.22)	1.07 (0.99–1.16)	1.00 (0.93–1.08)
**All diseases (A00–Y98)**	1.32 (1.25–1.39)	1.20 (1.17–1.23)	1.23 (1.21–1.26)	1.21 (1.19–1.23)

SMRs were estimated based on Chinese national mortality rates (not available before 1970).

The SMR from all causes was 0.83 (95% CI 0.80–0.85) among non-dust-exposed workers. In this group, we observed elevated SMRs for nasopharynx cancer (SMR 1.91, 95% CI 1.41–2.48), liver cancer (1.17, 1.04–1.31), hypertensive heart disease (2.24, 1.84–2.68), pulmonary heart disease (1.17, 1.04–1.32), and infectious diseases (1.98, 1.76–2.22), including respiratory tuberculosis (1.24, 1.08–1.41).

## Discussion

Our findings provide strong evidence that long-term silica dust exposure is associated with substantially increased mortality among Chinese workers. We not only confirmed significant relationships between increased silica dust exposure and heightened risk of death from respiratory diseases and lung cancer, but also found a significant exposure–response relationship between silica dust exposure and mortality from CVD, even at lower exposure levels.

These findings have important public health implications. Silica dust exposure is very common and is associated with increased morbidity and mortality from pneumoconiosis. Our study showed that the cumulative incidence of pneumoconiosis was 20.3% and the death rate from this disease in those with the disease was very high (61.7%). A report from the Chinese Ministry of Health indicated that the death rate from all reported pneumoconiosis was 23.1% between 1949 and 2008 [1]. Data from this study and prior ones provide strong evidence to support an association between silica dust exposure and increased mortality from cardiopulmonary diseases. We estimated that 4.2% of deaths (231,104) among industrial workers were attributable to dust exposure in China in 2008. It is well known that silica dust exposure is a preventable health hazard. These data underscore an urgent need to tighten regulations on dust control at worksites.

Dust exposure has been linked to risk of death in previous environmental and occupational health studies. The World Health Organization has estimated that 1.4% of all deaths result from exposure to various dust particles [23]. It is interesting that two cohort studies conducted in Germany among 17,644 porcelain production workers [24] and 19,943 construction workers [25] showed no significant increase in SMR from any cause. However, the mortality rates in both studies were very low, and the follow-up was relatively short. In addition, neither study determined levels of dust exposure. Our results are consistent with those from a study conducted among 3,010 tin miners in the UK (SMR 1.27) [26], although excess mortality in the UK cohort resulted from malignant neoplasms and accidents, not from respiratory diseases and CVDs. In our study, after 44 y of follow-up, we confirmed the adverse health effects of silica dust through exposure–response analysis of personal silica exposure and total mortality among 74,040 workers. After adjustment for potential confounders including cigarette smoking, silica dust exposure accounted for approximately 15.2% of all deaths in our cohort.

Our data suggest that silica dust substantially raises the risk of death from respiratory diseases as well as CVDs. Traditionally, non-malignant respiratory diseases were thought to be the main causes of death among dust-exposed workers [27]–[29]. However, this study showed that the proportion of deaths from respiratory diseases to all deaths decreased from 36.6% to 23.1%, while the proportion of deaths from diseases of the circulatory system increased from 29.4% to 37.9% from 1974 to 2003. Increased mortality from CVD was mainly due to higher rates of pulmonary heart disease from 1970 to 1974. Pulmonary heart disease was caused directly by high dust concentrations leading to a high prevalence of pneumoconiosis. From the second half of the 1960s onwards, there was a gradual decline in silica dust concentrations because of safer working practices and increased protective measures. From 1970 to 2003, the subtype of CVD changed: the proportion of deaths from pulmonary heart diseases decreased from 90.7% to 37.8%, while the proportion of deaths from hypertensive, ischemic, and chronic rheumatic heart disease increased from 5.4% to 41.3% during the same period. The SMRs of hypertensive, ischemic, and chronic rheumatic heart diseases gradually increased with ongoing follow-up of the cohort. Among workers exposed to respirable silica concentrations lower than 0.1 mg/m^3^ in their lifetime work histories, we observed elevated mortality from hypertensive heart disease, and ischemic heart disease. Among these workers, each 0.1 mg/m^3^-y increase in CDE was associated with a 2.2%, 6.0%, and 4.2% increase in the death rate from CVDs, pulmonary heart disease, and ischemic heart disease after adjusting for smoking and other confounder factors, respectively. These results indicate that low dust exposure is likely to contribute to CVDs without the presence of respiratory disease. Increased cardiovascular mortality in this study may be an independent and novel complication of silica exposure.

Several prior reports on the relationship between ambient particulate matter and cardiovascular mortality have focused on combustion-sourced particulate matter in cities [30],[31]. However, silica particles are not combustion-sourced. Rather, they are made up of a continuous framework of silicon–oxygen tetrahedral crystals, an essential constituent of granite and other felsitic igneous rocks. Increased risk of ischemic heart diseases from silica dust exposure was observed in South African gold miners and in the Swedish national census [32],[33], although these studies did not examine a dose–response relationship. Our study showed that non-combustion-sourced particles of crystalline silica were associated with elevated cardiovascular mortality, and this finding needs to be confirmed in further studies. The mechanisms by which non-combustion-sourced silica particulates increase the risk of CVD are largely unknown. Possibilities may involve the direct effects of fine particulates that cross the pulmonary epithelium into the cardiovascular system and lung receptors, or an indirect effect mediated through pulmonary oxidative stress and inflammatory responses [34]–[36].

In addition, we found elevated mortality from all causes, pneumoconiosis, infectious diseases, malignant neoplasms including nasopharynx cancer and liver cancer, and CVDs including ischemic heart disease and hypertensive heart disease among individuals who worked in an environment with respirable silica dust concentrations equal to or lower than 0.1 mg/m^3^. The 0.1-mg/m^3^ level is the exposure limit for respirable silica in the workplace specified by the US Occupational Safety and Health Administration. In China, the limit for respirable silica (0.07–0.35 mg/m^3^, depending on the percentage of silica dust) is similar to the US standard. However, even keeping silica exposure lower than 0.1 mg/m^3^ may not fully protect workers.

The association of silica dust exposure and lung cancer risk has been controversial for decades. In the present study, silica dust exposure was associated with lung cancer; risk ratios based on exposure levels ranged from 1.45 to 1.53. The penalized spline curve suggested a positive exposure–response association between silica exposure and lung cancer risk, although the HR decreased at higher levels of CDE. Possible explanations for the decrease in lung cancer risk at higher CDE include (1) a depletion of the number of susceptible workers in the cohort at high exposure levels and (2) bias introduced by the healthy worker survivor effect. This phenomenon was also observed in studies of other occupational populations [37]. Although adjustment for smoking did not change the overall shape of the exposure–response curve, it decreased the lung cancer mortality risk across the range of CDE levels, indicating a confounder effect of smoking in the association of silica exposure and lung cancer risk.

The strengths of this study include a large sample size, a long duration of follow-up, and a low rate of loss to follow-up (4.6%). We collected detailed information on silica dust exposure and cause-specific mortality; the diversity of mine types provided a wide range of exposures.

There were several limitations to this study. First, we did not collect data on dietary patterns and leisure time physical activity, and, therefore, we were unable to evaluate the confounding influence of these factors, especially on CVDs. However, diet and physical activity patterns were likely to be relatively homogenous in this cohort. Second, long-term exposure to silica dust was estimated carefully, but measurement errors were inevitable. Silica dust concentrations before 1950 were estimated using the concentrations in 1950, which may have led to underestimation of exposure for those who worked before 1950 (6,164 workers). Third, although the majority of deaths were ascertained by reviewing medical or accident records or death certificates, 4.3% of deaths were reported orally by relatives, yielding cause of death data that might not be reliable. However, results did not change after excluding these deaths. Finally, silica dust levels vary across different industries and companies, and thus the use of HRs estimated from this cohort may lead to inaccurate estimation of the PAR due to silica dust exposure for the entire population of Chinese industrial workers.

In summary, in this large cohort study, we found a significant exposure–response relationship between silica dust exposure and mortality from all causes, pneumoconiosis, and respiratory disease. Importantly, we also demonstrated a significant exposure–response relationship between silica dust exposure and CVDs. Findings from this study have important public health implications for improving occupational safety among those exposed to silica dust in China and around the world.

## Supporting Information

Figure S1 **Locations in China of the studied metal mines and pottery factories.**
(TIF)

Table S1 **Vital status of study individuals (** ***n*** **= 74,040) by the end of follow-up, according to type of mine/factory.**
(DOC)

## Abbreviations

CDE: cumulative silica dust exposure
CI: confidence interval
CVD: cardiovascular disease
HR: hazard ratio
ICD-10: 10th International Classification of Diseases
JEM: job–exposure matrix
PAR: population attributable risk
SMR: standardized mortality ratio
