# Supplementary figures and images for: Long-Term Exposure to Silica Dust and Risk of Total and Cause-Specific Mortality in Chinese Workers: A Cohort Study
Source: PLoS Med. 2012 Apr 17;9(4):e1001206. doi: 10.1371/journal.pmed.1001206 (PMC3328438; doi:10.1371/journal.pmed.1001206)

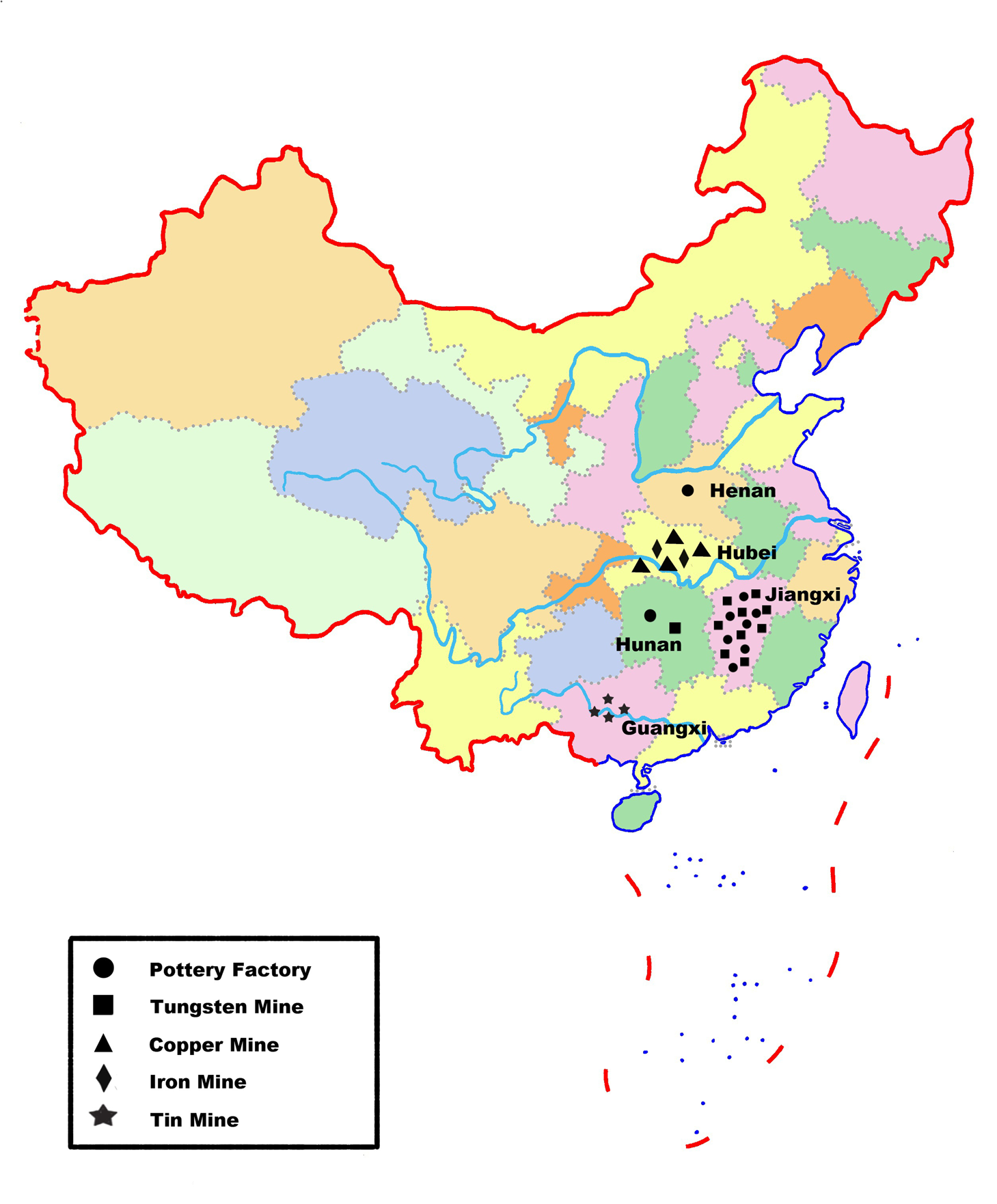

Supplement: Figure S1 — Locations in China of the studied metal mines and pottery factories. (TIF) [file pmed.1001206.s001.tif]
