# Supplementary material for: Long-Term Exposure to Silica Dust and Risk of Total and Cause-Specific Mortality in Chinese Workers: A Cohort Study
Source: PLoS Med. 2012 Apr 17;9(4):e1001206. doi: 10.1371/journal.pmed.1001206 (PMC3328438; doi:10.1371/journal.pmed.1001206)
Supplement: Table S1 — Vital status of study individuals ( n = 74,040) by the end of follow-up, according to type of mine/factory. (DOC) [file pmed.1001206.s002.doc]

**Table S1** Vital status of study subjects (*n* = 74,040) by the end of follow-up, according to types of mine/factory.

| **Vital Status** | **Tungsten Mines** | **Iron and Copper Mines** | **Tin Mines** | **Pottery Factories** | **Entire Cohort** |
| --- | --- | --- | --- | --- | --- |
| Working – no. (%) | 3033 (9.2) | 5103 (27.5) | 1633 (19.8) | 2238 (15.7) | 12007 (16.2) |
| Left – no. (%) | 2852 (8.7) | 3648 (19.6) | 1346 (16.3) | 1219 (8.5) | 9065 (12.2) |
| Retired – no. (%) | 16217 (49.3) | 7100 (38.2) | 3325 (40.3) | 6810 (47.6) | 33452 (45.2) |
| Deceased – no. (%) | 10816 (32.9) | 2731 (14.7) | 1939 (23.5) | 4030 (28.2) | 19516 (26.4) |

Percentages may not total 100 because of rounding.
